# Top(ological-operon) secret behind the long-range transcriptional coupling

**DOI:** 10.1038/s41392-022-01195-5

**Published:** 2022-10-15

**Authors:** Lei Zhang, Zhibing Wu, Huasong Lu

**Affiliations:** 1 Department of Orthopaedic Surgery, The First Affiliated Hospital of Wenzhou Medical University, Wenzhou, Zhejiang 325000 China; 2 Department of Oncology, Affiliated Zhejiang Hospital, Zhejiang University School of Medicine, Hangzhou Zhejiang, 310013 China; 3 Zhejiang Provincial Key Laboratory for Cancer Molecular Cell Biology, Life Sciences Institute, Zhejiang University, Hangzhou, Zhejiang 310058 China

**Keywords:** Molecular biology, Developmental biology

In a recent study published in *Nature*, Levo et al. reported that paralogous genes separated by long distances are regulated by specialized DNA stretches called tethering elements, which enable their physical associations and co-dependent transcriptional coupling during *Drosophila* embryogenesis.^1^

The eukaryotic genome is spatially organized via a hierarchy of multi-scale structures.^2,3^ Accumulating evidence suggests that the dynamic regulation of higher-order chromatin organization is mechanistically interesting and, more importantly, biologically significant. For instance, loop-like structure generated by cohesin complex facilitates the tethering of separated DNA elements into physical proximity for long-range gene regulation. Functionally, the proper organization of chromatin architecture plays an essential role in various biological processes such as lineage specification and cell differentiation, and disruption of these structures can lead to developmental disorders and human diseases.^3^ While the domain formation by loop extrusion mechanism has been extensively investigated to delineate the key events underlying promoter-enhancer communication and provide a foundation for understanding how distant regulatory elements act on corresponding individual genes to fine-tune their expression, other modes of regulatory interactions have been relatively understudied in the past. In particular, genes with long-range connectivity have been reported to be preferentially transcribed in a co-regulated manner.^4^ Yet, the causal relationship between spatial engagement of co-regulated genes and their transcriptional coupling remains debatable.

Paralogues are duplicated genes that reside at different genomic locations but play interconnected roles in a common biological process. Taking advantage of their overlapping expression pattern during embryonic development, Levo et al. explored the mechanistic principles underlying the coordinated expression of these genes. They first employed Micro-C, a state-of-art 3C-based method that provides finer-scale chromosome organization at nucleosome-resolution,^5^ for mapping chromatin folding in early fly embryos. This analysis revealed a large number of long-range focal contacts, most of which correspond to paralogues that are co-regulated by shared enhancers. These results therefore hinted that the conventional view of metazoan gene regulation, which claims that individual genes are independently controlled via their own regulatory elements, needs to be revisited to elucidate the co-dependent activation of these interconnected paralogues.

The authors then focused on two sets of paralogous genes, *knrl/kni* and *scyl/chrb*, for an in-depth characterization of their coordinated expression. They used CRISPR/Cas9-based targeted genome editing to insert structurally unique stem loops into the intronic region of respective genes, enabling the corresponding nascent transcript to be visualized by fluorescent coat proteins. This tagging strategy allowed the group to spatiotemporally evaluate the transcriptional activity in live embryos. Time-lapse quantitative analysis of nascent transcripts revealed that these distantly located paralogues became physically associated when transcription was turned on. They also exhibited higher co-initiation frequencies than uncoupled genes, supporting their transcriptional coupling during development.

Finally, the researchers sought to investigate the molecular underpinnings of synchronized gene expression. Owing to the enhanced resolution of Micro-C, the authors could unambiguously pinpoint the direct attachment sites between long-range connected gene pairs. The prominent feature of these promoter-proximal tethering elements is that they are physically segregated from the respective shared enhancers and are not bound by CTCF, indicating that they are likely formed in a CTCF-independent manner. Genetic perturbation in the tethering elements not only disrupted the spatial contacts between these genes, but also altered their co-dependent transcriptional bursting dynamics, presumably reflecting the destabilization of enhancer-promoter communications. Therefore, the authors concluded that promoter-proximal tethering elements support the promoter-anchored interactions between distant paralogues and contribute to their coordinated expression (Fig. 1).

**Fig. 1:**
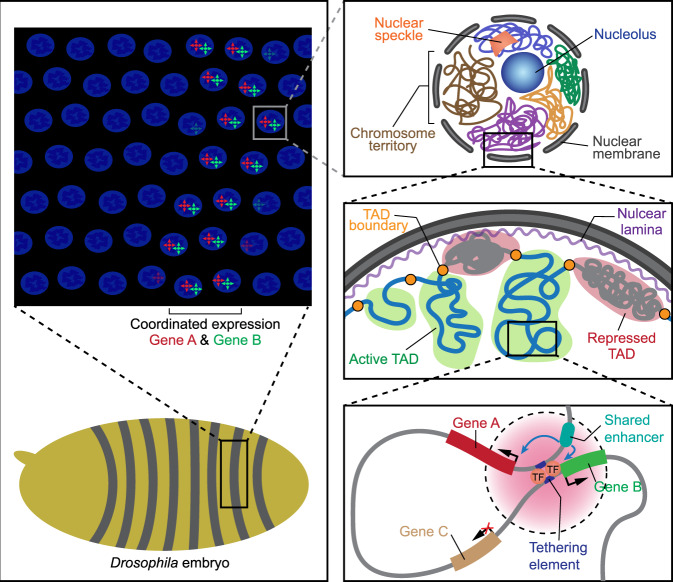
Topological operons for transcriptional coupling. Functionally linked paralogous genes are expressed in a co-dependent manner during embryonic development. Levo et al. have discovered that distant genes engaged in a common process are physically brought together in three-dimensional space by specialized genomic regulatory sequences called promoter-proximal tethering elements. This long-range interconnection between paralogous genes mediates the formation of co-transcriptional hubs and supports their coordinated expression required for the establishment of developmental patterns

Analogous to bacterial operons that provide a common control mechanism for simultaneous expression of functionally related genes to quickly adapt to environmental changes, eukaryotic paralogous genes involved in the same pathway are also co-regulated to ensure their synchronized activation in response to developmental cues. However, whereas bacterial genes falling under the same operon are physically close in the genome, eukaryotic paralogous genes might be separated by long linear distances. Reflecting this difference, the authors proposed the term “topological operon” to highlight the importance of higher-order chromatin organization for coordinated gene expression. In such eukaryotic operons, functionally related genes are not always in physical proximity, allowing the tethering elements to play a switch-like function to determine when and where the operons become activated. Moreover, the grouping of interconnected genes is no longer constrained by linear genomic distances in topological operons. This gives rise to the possibility that a given enhancer might be shared by different sets of genes because of the changes in 3D organization when cells are shifted from one developmental stage to another. Another possibility is that multiple tethering elements within the topological operon might have different degrees of tethering effect on gene-gene associations, thereby enabling distinct modes of co-regulation under different conditions. These intriguing prospects remain to be rigorously tested in the future.

This study also raises several other interesting questions. For example, what are the upstream signals that instruct the formation of topological operons? Are topological operons exclusively involved in developmental processes? Or can topological operons be reprogrammed in disease settings to promote aberrant gene expression? More importantly, what is the molecular grammar underlying the coordinated regulation of topological operons? Transcription factors such as GAF, CLAMP and Ph appear to be enriched at the promoter-proximal tethering elements.^1^ They may act in trans and function together with tethering elements to establish the long-range integration for transcriptional co-regulation. Nevertheless, the factors involved in the formation of topological operons remain to be identified. Finally, given the much larger size of mammalian genome than that of Drosophila, whether and to what extent the current findings can provide novel insights into the coordinated expression of functionally-related genes in mammalian cells is currently unclear. Thus, further studies on these crucial issues will be extremely informative in illuminating the detailed mechanisms of topological operon regulation.

In summary, this study sheds new light on the critical role of genome topology in the co-dependent expression of distant regulatory genes. It raises an interesting question: could this topological operon be the “top secret” underlying long-range transcriptional coupling? As technologies to map genome organization are constantly evolving, the answer to this query will undoubtedly be unveiled soon.

## References

[1] Levo M (2022). Transcriptional coupling of distant regulatory genes in living embryos. Nature.

[2] Gibcus JH, Dekker J (2013). The hierarchy of the 3D genome. Mol. Cell..

[3] Zheng H, Xie W (2019). The role of 3D genome organization in development and cell differentiation. Nat. Rev. Mol. Cell Biol..

[4] Zinani OQH, Keseroglu K, Ay A, Ozbudak EM (2021). Pairing of segmentation clock genes drives robust pattern formation. Nature.

[5] Hsieh TS (2020). Resolving the 3D landscape of transcription-linked mammalian chromatin folding. Mol. Cell..

